# Failure to Thrive: Nursing Home Staff Experiences in Caring for Residents during the COVID-19 Pandemic

**DOI:** 10.1093/geroni/igab046.3193

**Published:** 2021-12-17

**Authors:** Yu-Ping Chang, Audrieanna Raciti, Cristina de Rosa, Margaret Doerzbacher, Yanjun ZHou, Chia-Hui Chen, Amy Lyons

**Affiliations:** 1 University at Buffalo, Buffalo, New York, United States; 2 University at Buffalo, 7168292015, New York, United States

## Abstract

Nursing home residents and staff have accounted for roughly 40% of Coronavirus-related deaths in the U.S. The burden of caring for vulnerable residents coupled with isolation policies has taken a significant emotional toll among direct health care staff in long term care facilities. This study explores nursing home staff’s experiences in caring for residents during the COVID-19 pandemic. A qualitative descriptive approach with a semi-structured guide was used to conduct individual interviews. We recruited nursing home staff employed during the COVID-19 pandemic in long term care facilities located in New York State. Interviews were recorded, transcribed verbatim, and then analyzed using Braun and Clarke’s Reflexive Thematic analysis. Twelve nursing home staff were interviewed. Participants consistently refer to failure-to-thrive as an extremely concerning problem because many residents demonstrate decreased appetite and poor nutrition, inactivity, and depressive symptoms due to social isolation. They also often feel frustrated and overwhelmed due to uncertainty and shortages of staff. Five main themes were identified, including doing their best to manage residents’ failure-to-thrive, working as a team, keeping family members informed and connected, struggling to balance competing personal and professional demands, and needing support to reduce stress and build strength. Our study findings indicate that nursing home staff experienced a high level of stress and identified failure-to-thrive caused by isolation and loneliness as a common phenomenon among nursing home residents during the COVID-19 pandemic. Interventions are urgently needed to reduce isolation and loneness in nursing home residents and to provide support for staff.

